# miR-224-3p inhibits autophagy in cervical cancer cells by targeting FIP200

**DOI:** 10.1038/srep33229

**Published:** 2016-09-12

**Authors:** Wang Fang, Shan Shu, Li Yongmei, Zhu Endong, Yin Lirong, Sun Bei

**Affiliations:** 1 The Second Hospital of Tianjin Medical University, 300070 Tianjin, China; 2 Department of Medical Microbiology, School of Basic Medical Sciences, Tianjin Medical University, 300070 Tianjin, China; 3 Key Laboratory of Hormones and Development (Ministry of Health), Metabolic Diseases Hospital & Tianjin Institute of Endocrinology, Department of physiology, Tianjin Medical University, 300070 Tianjin, China

## Abstract

Cervical cancer (CC) is a malignant solid tumor, which is one of the main causes of morbidity and mortality in women. Persistent High-risk human papillomavirus (hrHPV) infection is closely related to cervical cancer and autophagy has been suggested to inhibit viral infections. miRNAs have been reported to regulate autophagy in many solid tumors with many studies implicating miR-224-3p in the regulation of autophagy. In this study, we performed a miRNA microarray analysis on CC tissues and found that a large number of miRNAs with differential expressions in hrHPV-infected tissues. We identified miR-224-3p as a candidate miRNA selectively up regulated in HPV-infected tissues and cell lines. Further analysis revealed that miR-224-3p regulates autophagy in cervical cancer tissues and cell lines. While the overexpression of miR-224-3p inhibits autophagy in HPV-infected cells, knocking down endogenous miR-224-3p increases autophagy activity in the same cells. In addition, we found that miR-224-3p directly inhibits the expression of autophagy related gene, FAK family-interacting protein of 200 kDa (FIP200). In summary, we found that miR-224-3p regulates autophagy in hrHPV-induced cervical cancer cells through targeting FIP200 expression.

Cervical Cancer (CC) is a common gynecological malignancy and a leading cause of cancer-associated mortality in females, especially in developing countries^1^. In China, the incidence of cervical cancer is on the rise with a younger median age at diagnosis. Although studies have shown that high-risk human papillomavirus (hrHPV) can be detected in almost all types of HPV-induced cervical cancer^2^,^3^, pathogenesis of the disease remains poorly understood.

The viral oncogenes *E6* and *E7*, especially from the high-risk HPV-16 or/and HPV-18 subtypes are the main mediators of HPV-induced tumorigenesis and play key roles in CC pathogenesis^4^,^5^. Expression of these oncoproteins result in the loss of function of host tumor-suppressor proteins, causing abnormalities in cell cycle regulation and host-cell biological activity. This in turn increases transformation, invasion and metastatic potential of host cells. Although *E6* and *E7* oncogene activity has been widely recognized as the main pathological factors behind HPV-induced CC, recent studies have shown that hrHPV can also inhibit autophagy in host cells^6^. However, our understanding of the molecular mechanisms by which hrHPV regulates autophagy in cervical cancer is still limited.

Autophagy is a highly conserved multi-step lysosomal degradation process where cellular components are sequestered in autophagosomes and subsequently fuse with lysosomes to degrade the sequestered contents. Recent studies suggest a close relationship between autophagy and tumor progression, and the dynamics of this relationship changes constantly throughout disease progression^7^. This dynamic role of autophagy in tumor progression has kindled much interest in the development of therapeutics that targets the autophagy pathway as a cancer treatment strategy. Interestingly, it was also recently suggested that host cells could utilize the autophagy process to protect against viral infections^8^. This exciting finding leads us to hypothesize that molecular interventions that increase cellular autophagy levels could enhance host cell resistance to hrHPV infections. A clearer understanding of the molecular underpinnings of how hrHPV infection regulates host cell autophagy will enable us to better design molecularly targeted therapeutics which can promote host resistance to hrHPV infections and subsequent progression to CC.

Autophagy-related genes (ATGs) are essential drivers of autophagy and are directly regulated by multiple microRNAs (miRNA)^9^. miRNAs are a set of non-coding RNAs that are initially reported to play a role in transcription regulation. Many studies have shown the involvement of miRNAs in the control of key pathways involved in different physiological and pathological process^10^. miRNAs control a large number genes that affect cellular processes including proliferation, differentiation and apoptosis and their involvement in cancer pathogenesis has been extensively studied. A growing body of evidence is implicating miRNAs in the regulation of autophagy and further studies into the role of miRNAs in the control of autophagy will expand our knowledge on the molecular mechanisms behind miRNA-mediated autophagy regulation.

It has recently been suggested that miRNAs present in cells may participate in host–virus interactions influencing viral replication^11^. The mechanism of CC development has yet to be fully elucidated, but its pathogenesis is closely related to hrHPV infection. Although research has shown that hrHPV does not express miRNAs^12^. it has been proposed that hrHPVs can increase the expression of host-cell oncogenic miRNAs or down-regulate tumor suppressive miRNAs in cervical cells^13^,^14^,^15^.

Given the proposed role of miRNAs in the regulation of autophagy and the relationship between autophagy and resistance to viral infection, we conducted miRNA expression profiling in hrHPV cervical cancer tissues with the aim to identify unique autophagy related miRNAs which expression will be associated with hrHPV infection in cervical cancer. We also sought to characterize the mechanism behind how the dysregulation of miRNA expression could affect host cell autophagy levels. Persistent hrHPV infections is the major contributor to CC progression and increasing the host cells’ ability to clear hrHPV infection through augmenting autophagy in these cells is an exciting new avenue which should be explored in CC preventive therapy.

## Results

### hrHPV infection is associated with increased levels of miR-224-3p expression and decreased autophagy levels in cervical lesions

**Figure 1:**
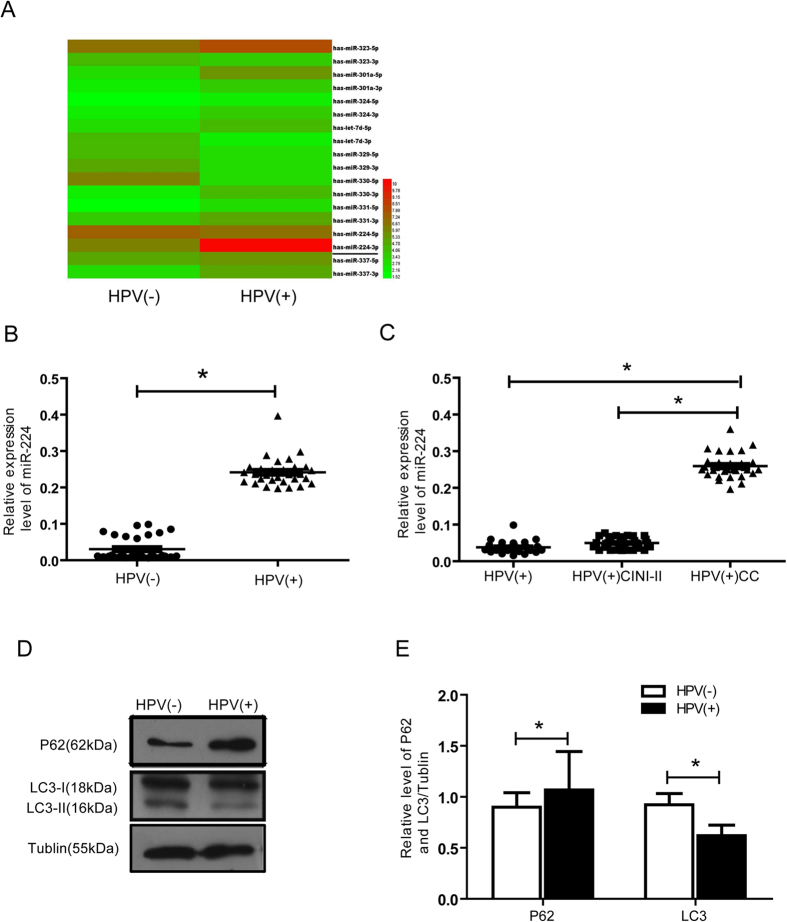
miR-224-3p is up regulated in HPV (+) cervical tissues and this correlates with lowered levels of autophagy. (**A**) The cervical tissues were collected for miRNA microarray analysis. A portion of the heat map of analyzed miRNAs is shown. Highly up-regulated miRNAs are shown in red, while down-regulated microRNAs are shown in green. miR-224-3p is underlined. (**B**) Collected tissues analyzed for miR-224-3p expression using qPCR. (**C**) miR-224-3p expression in cervical lesions at different stages was determined by qPCR analysis. (**D**,**E)** p62 and LC3-II levels were analyzed using lysates extracted from human cervical tissues. Expression levels of each protein relative to tubulin are represented as a bar graph in (**E**) Data is represented as mean ± SD of *n* = 3 independent experiments. The boxes represent the lower and upper quartile and median; whiskers illustrate the 10th to 90th percentiles of the samples. *p < 0.05, as determined by student’s 2-tailed *t-*test or one-way ANOVA.

To identify candidate miRNAs whose expression levels are altered in association with hrHPV infection, we conducted a miRNA microarray screen using 150 clinical samples collected from patients diagnosed with various stages of HPV-induced cervicitis as well as from patients with non-HPV-associated cervicitis. In this initial screen, we identified hypoxia-induced autophagy related miR-224-3p as the miRNA that is selectively up regulated in HPV-positive samples (Fig. 1A). Independent validation using quantitative PCR further confirmed this observation (Fig. 1B). To further correlate miR-224-3p expression to CC staging, we subcategorize the HPV-positive group into HPV (+), HPV (+) CIN I-II and HPV (+) CC. In this analysis, we found that as the level of malignancy increases there is a concurrent up-regulation of miR-224-3p expression, with the highest expression observed in the HPV (+) CC samples (Fig. 1C).

To determine if this up-regulation of miR-224-3p had any observable effects on autophagy levels, we next sort to compare the expression levels of known autophagy markers, LC3-II^16^ and p62^17^ in HPV-positive versus HPV-negative samples. In western blotting analysis, we noted a marked increase in p62 levels in HPV-positive tissue compared to HPV-negative tissue while LC3-II levels was observed to decrease significantly in HPV-positive tissue (Fig. 1D,E), indicating a drop in autophagy levels in HPV-positive tissue. Taken together, these data suggest that hrHPV infection results in miR-224-3p up-regulation and this is accompanied by a decrease in autophagy levels in HPV-positive tissue.

### hrHPV-positive cervical cancer cell lines express high levels of miR-224-3p and this is linked to the reduced levels of autophagy

**Figure 2:**
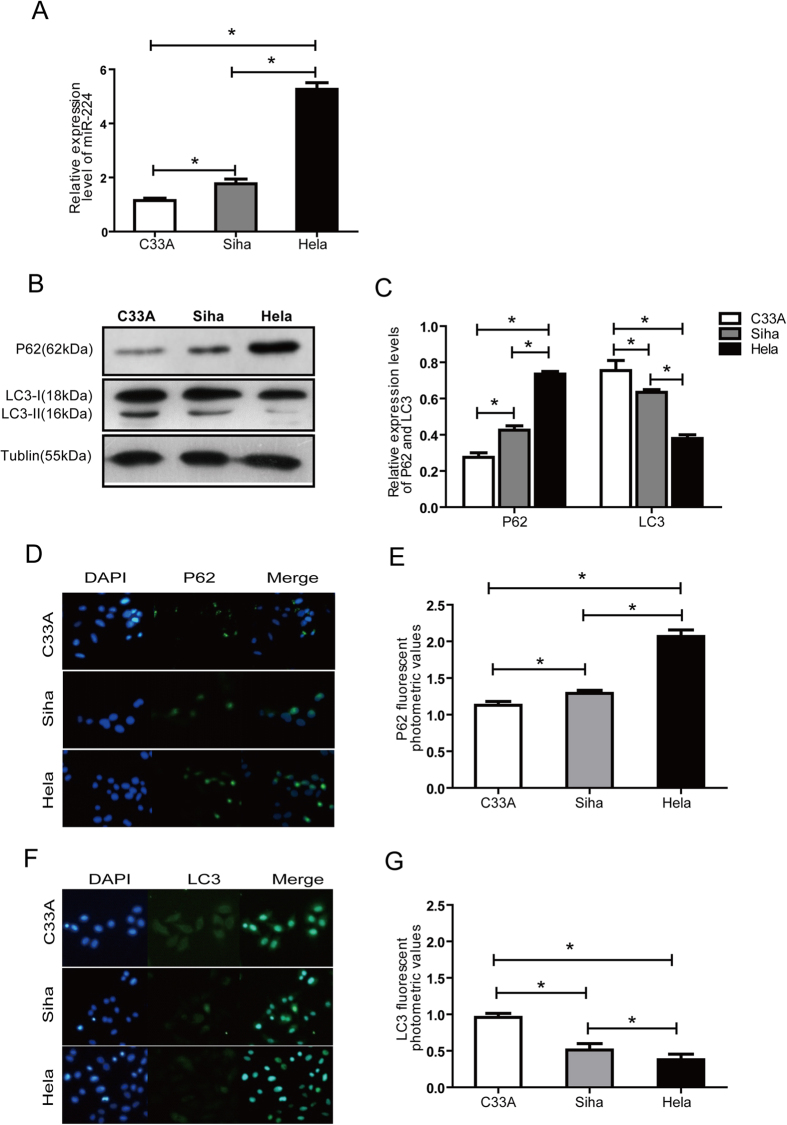
miR-224-3p induces autophagy in HPV(−)/(+) cervical cancer cells. (**A**) Cells harvested for miR224-3p expression quantification using qPCR. (**B**,**C**) C33A, SiHa and HeLa cells were cultured under the same conditions. LC3-II, p62 (SQSTM1) and tubulin levels were determined by Western blot. The expression levels of LC3-II and p62 were normalized to tubulin. (**D**,**E**) p62 levels in the C33A, SiHa and Hela cells were analyzed via immunofluorescence. Nuclei were stained using DAPI. Representative images are shown. (**F**,**G**) LC3-II levels in the C33A, SiHa and HeLa cells were analyzed via immunofluorescence. Nuclei were stained by DAPI. Representative images are shown. The data shown are represented as mean ± SD of. *n* = 3 independent experiments. *p < 0.05 as determined by one-way ANOVA.

To study the molecular mechanisms linking miR-224-3p expression to autophagy levels in HPV-associated CC, we next validated the association observed in clinical samples in HPV-positive and -negative cell line models which will be used in our mechanistic studies. For this purpose, we chose HPV-negative C33A cells, HPV16-positive SiHa cells and HPV18-positive HeLa cells as our cell line models. Similar to the observations in clinical samples, miR-224-3p expression was found to be significantly higher in HPV16/18-postive SiHa and HeLa cells when compared to HPV-negative C33A cells. Our quantitative PCR results revealed that the expression of miR-224-3p was lowest in HPV-negative C33A while miR-224-3p expression in HeLa cells was higher than that of SiHa cells (Fig. 2A). Both western blotting and immunofluorescence staining for autophagy markers p62 and LC3-II in these cells were used to detect the levels of autophagy. In these assays, we observed that the autophagy activity of HeLa cells was the lowest, while C33A cells showed the highest autophagy levels, evidenced by the accumulation of p62 and loss of LC3-II expression. The P62 was upregulated and the LC3-II was down regulated in the western-blot (Fig. 2B,C). The P62 was upregulated (Fig. 2D,E) and the LC3-II was down regulated (Fig. 2F,G) in the immunofluorescence. These observations further suggest that high miR-224-3p expression is selectively observed in HPV-positive cells and points to its possible role in the inhibition of autophagy in these cells.

### miR-224-3p expression in cervical cancer cells is directly linked to autophagy levels in these cells

**Figure 3:**
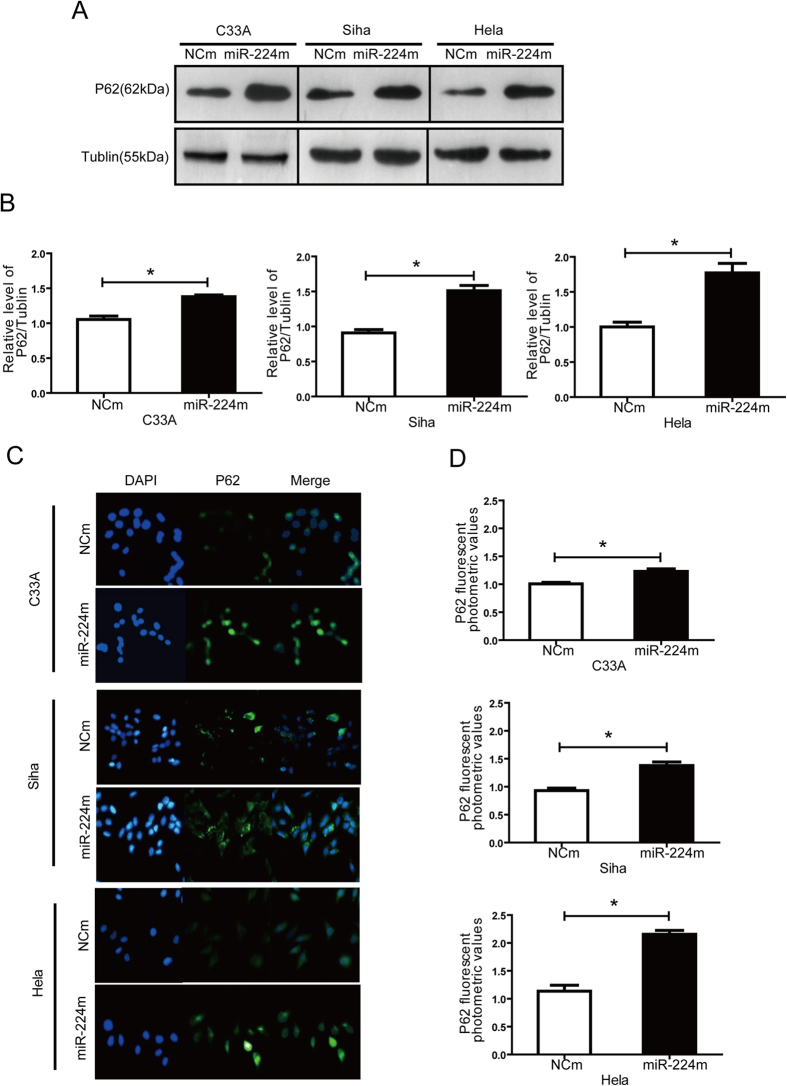
Ectopic expression of miR-224-3p inhibits autophagy in cervical cancer cells regardless of HPV status, but is more prominent in HPV(+) cells, especially in HPV18(+) cells. (**A**,**B**). C33A, SiHa and HeLa cells were transfected with miR-224-3p mimicsor NCm (100 nM) for 24 h. p62 and tubulin levels were determined by Western blot. (**C,D**). C33A, SiHa and HeLa were treated with miR-224-3p mimicsor NCm (100 nM) for 24 h and fixed. p62 levels were analyzed by immunofluorescence. Nuclei were stained with DAPI. Representative images are shown. Data is represented as mean ± SD of *n* = 3 independent experiments. *p < 0.05 as determined by student’s 2-tailed *t-*test.

To link miR-224-3p expression to autophagy levels we next ectopically expressed miR-224-3p using miR-224-3p mimics(miR-224m) in C33A, SiHa and HeLa cells and studied its effect on autophagy in these cells. Using the accumulation of p62 as a readout, it was observed in both western blotting (Fig. 3A,B) and immunofluorescence assay (Fig. 3C,D) that the introduction of miR-224m into all three cell lines induced a marked accumulation of p62 levels compared to the NCm transfected controls.

**Figure 4:**
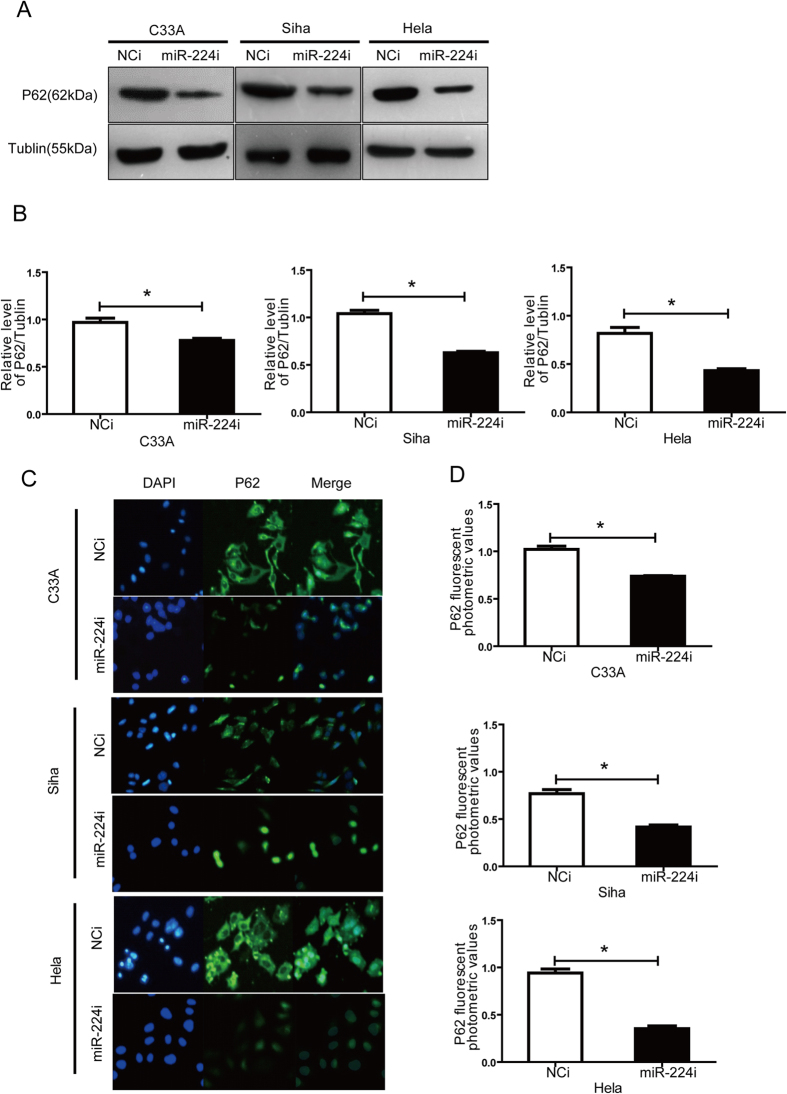
Inhibition of miR-224-3p activity increases autophagy levels in cervical cancer cells regardless of HPV status, but is more prominent in HPV(+) cells, especially in HPV18(+) cells. (**A**,**B**) C33A, SiHa and HeLa cells were transfected with miR-224-3p inhibitor or NCi (100 nM) for 24 h. p62 and tubulin levels were determined by Western-blot. (**C**,**D**) C33A, SiHa and HeLa cells were treated with miR-224-3p inhibitor or NCi (100 nM) for 24 h and fixed. p62 levels were determined by immunofluorescence. Nuclei were stained using DAPI. Representative images are shown. Data is represented as mean ± SD of *n* = 3 independent experiments. *p < 0.05 as determined by student’s 2-tailed *t-*test.

To further investigate the effect of miR-224-3p on autophagy, endogenous miR-224-3p activity was inhibited via the use of miR-224-3p inhibitors. Anti-miR designed against miR-224-3p (miR-224i) was transfected into C33A, SiHa and HeLa cells and p62 expression was measured by western blotting (Fig. 4A,B) and immunofluorescence (Fig. 4C,D). The miR-224-3p inhibitor down-regulated p62 expression in all three cell lines suggesting that miR-224-3p inhibition enhanced cellular autophagy levels in these cells. Taken together, our overexpression and inhibition studies directly links the expression and activity of miR-224-3p to cellular autophagy levels in cervical cancer cells. It is also noteworthy that in both ectopic expression and inhibition studies, the effects of miR-224-3p expression on cellular autophagy levels was more pronounced in HPV-positive SiHa and HeLa cells compared to HPV-negative C33A cells. This could suggest that HPV-infected cells are more sensitive to changes in miR-224-3p expression and could be more amenable to therapeutics that target miR-224-3p-mediated autophagy control.

### miR-224-3p inhibits autophagy through its regulation of autophagosome protein FIP200

**Figure 5:**
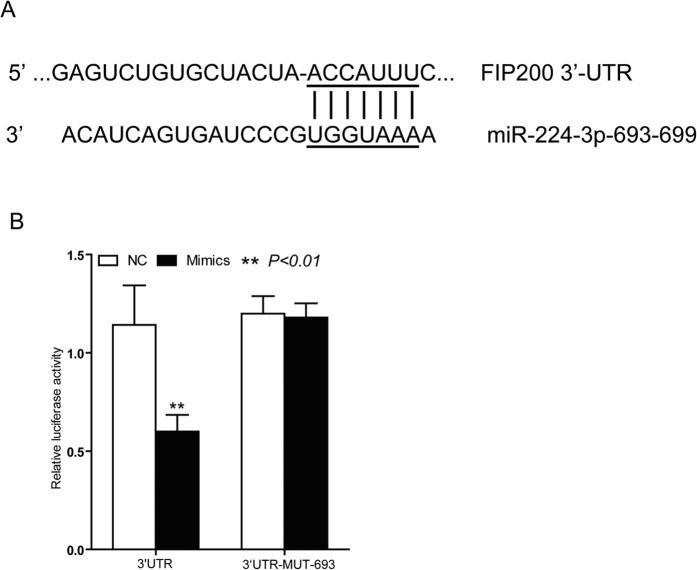
FIP200 is a target of miR-224-3p. (**A**) Predicted binding sequences for miR224-3p in the FIP200 3′-UTR. (**B**) Dual-luciferase reporter vectors containing the wild-type or mutated 3′-UTR fragments of FIP200 cloned into the pmirGLO-luciferase plasmids. Luciferase activity was assayed 48 h after co-transfection with either wild-type (WT) or mutated (MUT) 3′-UTR containing plasmids and miR224m or NCm in HeLa cells. Data is represented as mean ± SD of *n* = 3 independent experiments. miR-224m = miR-224-3p mimetic; NCm = miRNA mimicsnegative control. *p < 0.05 as determined using student’s 2-tailed *t-*test.

In order to elucidate the molecular mechanisms behind miR-224-3p-mediated autophagy control, we next decided to identify potential targets in the autophagy pathway that could be regulated by miR-224-3p. Using Target Scan 6.3, a web server that predicts biological targets of miRNAs developed by the Bartel laboratory at the Whitehead Institute for Biomedical Research, miR-224-3p was identified as a potential regulator of FIP200, a protein essential for autophagosome formation via its interaction with ULK^18^. Target Scan analysis predicted two putative miR-224-3p binding site within the 3′ UTR of FIP200 (Fig. 5A). To determine if miR-224-3p could effectively regulate FIP200 expression through its interaction with the FIP200 3′ UTR, we utilized a dual-luciferase reporter system where either the wild-type 3′ UTR or a mutated 3′ UTR (where the predicted miR-224-3p binding site is mutated such that miR-224-3p fail to bind) of FIP200 was cloned upstream the luciferase reporter gene. In this reporter system, we observed that ectopic expression of miR-224-3p mimics (miR-224m) reduced the level of luciferase activity in the wild-type 3′ UTR reporter but had no effect on the luciferase activity of the mutant 3′ UTR reporter. These results confirmed FIP200 as a direct target of miR-224-3p (Fig. 5B).

**Figure 6:**
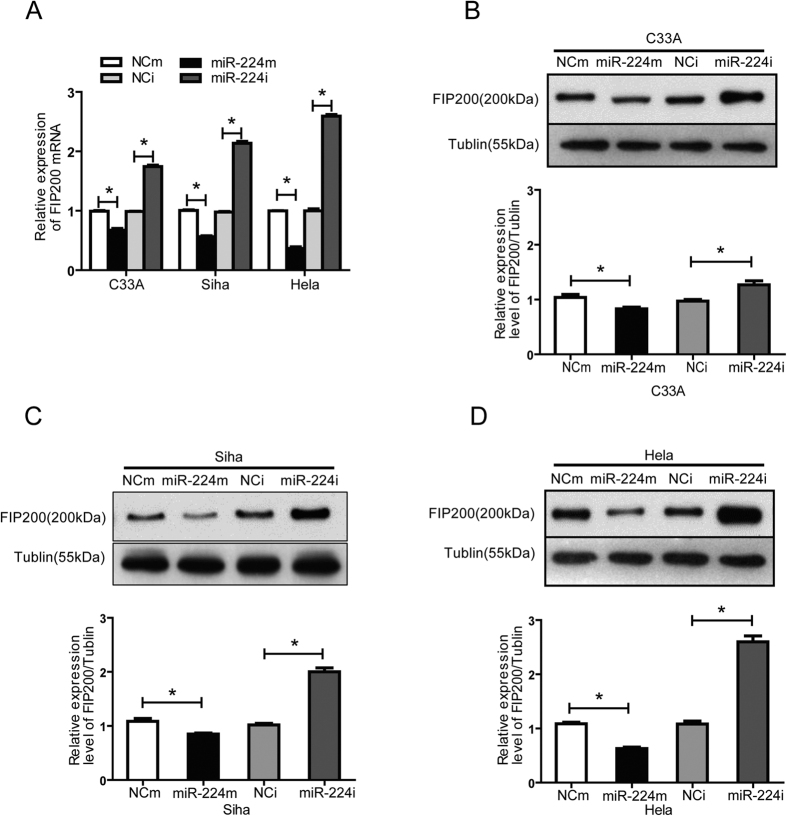
miR-224-3p regulates autophagy through FIP200 down regulation. (**A**,**B**) Levels of FIP200 and tubulin in C33A, SiHa and HeLa cells. Cells were transfected with miR-224-3p mimetic, NCm, miR-224-3p inhibitor or NCi (100 nM) for 24 h. Levels of FIP200 and tubulin were determined by Western blot. (**C**) The impact of miR-224-3p activation or inhibition on FIP200 mRNA levels were compared C33A, SiHa and HeLa cells. Data is represented as mean ± SD of *n* = 3 independent experiments. miR-224m = miR-224-3p mimetic; NCm = miRNA mimicsnegative control. *p < 0.05 as determined using student’s 2-tailed *t-*test.

Next to validate the role of miR-224-3p in the regulation of FIP200 expression *in vivo*, we examined the protein levels of FIP200 in C33A, SiHa and HeLa cells after treatment with the miR-224-3p mimics(miR-224m) or the miR-224-3p inhibitor (miR-224i). In all three cell lines, enforced miR-224-3p expression significantly decreased FIP200 expression while miR-224-3p inhibition increased FIP200 expression at both the mRNA and protein levels (Fig. 6). Taken together, these results suggest that miR-224-3p can directly affect autophagy levels in CC cells via its regulation of FIP200 expression.

## Discussion

Autophagy is a complex process that can be regulated by multiple pathways and induced by a wide variety of factors such as growth factors, amino acids, changes in glucose levels and DNA damage. Pathologically, autophagy is also linked to many diseases such as chronic inflammation, autoimmune disease and diabetes. In particular, autophagy has been frequently linked to cancer pathogenesis with many tumors observed to have dysregulated autophagic activity^19^,^20^. The widespread involvement of this complex cellular degradation process in the pathology of so many diseases highlights its potential as a target pathway for the development of molecularly targeted-therapeutics in disease management.

Although the role of autophagy in cancer progression has been extensively studied in the recent years, the involvement of autophagy in cervical cancer pathogenesis is less clear. With the increased incidence of HPV-induced cervical cancer due to chronic hrHPV infection in developing nations, there is a pressing need for the identification of novel molecular targets that can be used for the development of novel therapeutics or as biomarkers of hrHPV infection for cervical cancer preventive therapy.

Recent studies have indicated that hrHPV infection in cervical lesions and cervical cancer cell lines can specifically augment the expression levels of miRNAs^21^,^22^. In normal physiological conditions, miRNA expression seems to show tissue specificity and in our study, we report that expression of specific miRNAs can change as hrHPV-induced cervical lesion progress from benign to malignant. This finding highlights the significance of mapping the expression changes of miRNAs in hrHPV-associated cervical cancer progression for the identification of miRNA targets, which could be used as biomarkers, or therapeutic targets in the diagnosis and treatment of hrHPV-related cervical lesions. Current studies have identified miR-224-3p as the most common microRNA whose expression is dysregulated in malignant tumors^23^,^24^. Reports have indicted that miR-224-3p can promote the proliferation and metastasis of lung cancer cells by inhibiting TNF-α-induced apoptosis^25^. Apart from its role in lung cancer, miR-224-3p is also found to be over-expressed in hepatocellular carcinoma^26^. In cervical cancer, reports have shown that the expression of miR-224-3p in cervical cancer tissue is higher compared to surrounding normal tissue^27^. Moreover, up-regulated expression of miR-224-3p is closely linked to cervical cancer progression and poor prognosis^28^,^29^.

In this current study, we identified miR-224-3p as the miRNA whose expression was increased as cervical lesions progress through the different stages of malignancy. In our analysis we further identified that this correlation between miR-224-3p expression and cervical cancer stage was more closely linked to HPV status of the cervical lesion, with HPV-positive cells displaying significantly higher levels of miR-224-3p compared to HPV-negative cells. This exciting new finding prompted us to further investigate the mechanism behind hrHPV-induced cervical cancer progression in relation to miR-224-3p expression up-regulation and its effects on cellular pathways leading to cervical cancer pathogenesis.

MicroRNAs have diverse roles in cellular biology and is involved in the regulation of a wide variety of cellular pathways. Interestingly, the same cellular pathways that it regulates could also regulate the expression of miRNAs. An example will be autophagy where it has been shown that miRNAs can regulate the levels of autophagy in cells while autophagy levels can in turn regulate the expression of certain miRNAs^30^. Many studies have found that miRNAs can augment autophagy in malignant tumors, and this change affects tumor resistance to certain therapeutic agents^31^. With the increasing body of evidence suggesting that miRNA-regulated autophagy is a key occurrence in tumor progression, elucidation of the molecular mechanisms behind this regulation is quickly gaining recognition as a crucial gap which needs to be address in our understanding of cancer pathogenesis. In HPV-induced cervical cancers, persistent hrHPV infection has long been known to be a key contributor to transformation. Recent studies that suggest that virus have the ability to augment cellular autophagy levels led us to investigate the link between hrHPV infections, miRNA expression and autophagy.

To better understand the effects of miR-224-3p on autophagy in cervical cancer progression, we first looked at miR-224-3p expression across HPV-negative and positive cervical lesions. In our initial screen we found that miR-224-3p was selectively upregulated in hrHPV-positive cervical lesions and its expression levels was closely correlated to the level of tumor malignancy. In our study, we also report that hrHPV-induced cervical cancer cells expressed high levels of miR-224-3p and this correlates to a decrease in cellular autophagy levels in these cells. In over-expression and inhibition studies we successfully showed that miR-224-3p levels and activity directly affects cellular autophagy levels. We further identified the autophagosome formation-essential protein FIP200 as a direct gene target of miR-224-3p.

With the recent finding that viruses are able to augment host cell autophagy levels to support its survival, the link our study identified between miR-224-3p, its role in inhibiting host cell autophagy and hrHPV-infection in cervical lesions provide novel insights into how the human papillomavirus could alter host cell cellular autophagy to support its maintenance. Our data is the first to highlight the relationship between hrHPV-infection and miR-224-3p up-regulation. We are also the first to show that miR-224-3p regulates autophagy in hrHPV-induced cervical cancer cells through its action as a direct repressor of FIP200. Our findings open up new avenues for the use of miR-224-3p and FIP200 as novel biomarkers or molecular targets in the development of diagnostic and therapeutic strategies for hrHPV-induced cervical cancer.

## Materials and Method

### Tissue samples and miRNA microarray

Surgical cervical tissue samples were collected from 150 cases of clinically diagnosed cervical lesions. All collected samples were filtered using clinical medical records, clinical staging and pathological type. Only samples from patients with no prior medical therapy were finally selected for our analysis. Selected samples were subcategorized into HPV (+)/(−) normal cervical tissue, HPV (+) CIN I - II, HPV (+) CINIII grade and cervical squamous cell carcinoma. Protein lysates from the human tissues using cell lysis solution (5 μl RIPA + 50 μl PMSF), and supernatant collected after centrifugation. The protein was stored at −80 °C after protein concentration was determined via Bradford protein quantification method using a BSA standard curve. The MicroRNA microarray gene expression experiments and data analysis were conducted by Shanghai Biotechnology Co., Ltd.

### Cell culture

The human cervical cancer cell lines (C33A, Siha, Hela) were purchased from the Chinese Academy of Sciences Cell Bank (Shanghai, China). Cells were cultured in alpha-MEM with heat-inactivated 10% FBS, 100 U/ml penicillin and 100 g/ml streptomycin at 37 °C in a humidified incubator containing 5% CO_2_.

### Analysis of miR-224 expression by q-PCR

Total RNA was isolated by using Trizol reagent (Invitrogen, Carlsbad, CA, USA). Total RNA (1 μg) was reverse-transcribed with miR-224-3p, stem-loop and U6 snoRNA RT primers (Sangon Biotech, Shanghai, China) by using a RT-PCR kit (AT301 TransGen Biotech, Beijing, China) according to the manufacturer’s protocol for cDNA synthesis. The TransStart Top Green qPCR SuperMix kit was used for qPCR (AQ131, TransGen Biotech, Beijing, China). Real-time PCR was performed by using a SGExcel FastSYBR Mixture kit (B532955-0001, Sangon Biotech, Shanghai, China) with miR224-3p and U6 snoRNA primers. mRNA levels were normalized to GAPDH. U6 expression was used as the endogenous control for miRNA level. Expression analysis was calculated using the comparative Ct method.

### Western blot analysis

The protein from the cervical tissues and cell extracts were resolved by SDS-PAGE, and analyzed by western blotting. Antibodies used for western blotting were as follows: SQSTM1/P62(Abcam, ab91526), LC3A/B(Abcam, ab128025), FIP200(Abcam,ab176816). Following incubation with horseradish peroxidase-coupled secondary anti-mouse/rabbit (Tianjin Sungene Biotech), protein bands were visualized using ECL Blotting Detection Reagents (NCI4106, Thermo Pierce ECL).

### MiRNAs and transfections

The synthetic miRNAs (miR-224-3p), miR-224-3p-inhibitor, miR-mimics N.C. and the miR-inhibitor N.C. were purchased from Shanghai Gene Pharma Co. Ltd. The cervical cancer cell lines were transiently transfected using lipofectamine™ 2000 (Invitrogen,11668-019) according to the manufacturer’s protocol. The access number of miR-224-3p is MIMAT0009198 and the FIP200 is NM_014781.4. The sequences of miRNA (miR-224-3p) mimics, inhibitors, negative control and miRNA inhibitor N.C are as follows:

miR-224-3p mimics: 5′-AAAAUGGUGCCCUAGUGACUACA-3′,

miR-224-3p inhibitor: 5′-UGAAGUCACUAGGGCACCAUUUU-3′,

NCm: 5′-UUCUCCGAACGUGUCACGUTT-3′,

NCi: 5′-CAGUACUUUUGUGUAGUACAA-3′.

### Immunofluorescence and Confocal imaging

The three cell lines were transfected the miR-224-mimics/inhibitor as described above. Cells were fixed in 4% paraformaldehyde and permeabilized by 1% Triton X-100. Following blocking with 1% bovine serum albumin, cells were serially incubated in rabbit anti-LC3 (Abcam) and Goat anti-rabbit Alexa Fluo488 (Invitrogen). The images were acquired using the AV300-ASW confocal microscope (Olympus America Inc., Center Valley, USA) with a 60 × oil lens. The pictures analysed using Image-Pro Plus 6.0 (Media Cybernetics)^32^.

### Target prediction and luciferase reporter assay

Avaliable online tools, Targetscan (http://www.targetscan.org) and Findtar (http://bio.sz.tsinghua.edu.cn) were used to predict the miR-224-3p targets. The targets predicted by these 2 programs were further analyzed and demonstrated by the following biological experiments. For FIP200, a 290-bp fragment of the 3′ UTR sequence containing two putative miR-224-3p binding sites was cloned into the pmirGLO-luciferase reporter plasmids. CC cells were co-transfected with the luciferase reporters together with miR-224-3p mimics, miR-N.C., miR-224-3p inhibitor and miR-inhibitor-N.C. by using Lipofectamine 2000.

### Statistical analyses

All data were analyzed by SPSS 16.0 (SPSS, IL, USA) and GraphPad-Prism5 (Graph Pad, CA, USA) in one-way ANOVA, Student’s 2-tailed t-test or Fisher’s exact test. Data are showed as mean ± standard deviation (SD) of three independent experiments, followed by Dunnett’s test for multiple comparisons of the means. All tests were 2-tailed, and p < 0.05 was statistically significant.

### Ethics statement

We confirm that all experiments were performed in accordance with relevant guidelines and regulations. The research was conducted in accordance with the Declaration of Helsinki. Ethical approval was given by the medical ethics committee of the The Second Hospital of Tianjin Medical University with the following reference number: KY2016K016. We confirm that informed consent was obtained from all subjects.

## Additional Information

**How to cite this article**: Fang, W. *et al*. miR-224-3p inhibits autophagy in cervical cancer cells by targeting FIP200. *Sci. Rep.* **6**, 33229; doi: 10.1038/srep33229 (2016).
